# Angiosarcoma treated successfully with anti-PD-1 therapy - a case report

**DOI:** 10.1186/s40425-017-0263-0

**Published:** 2017-07-18

**Authors:** Simran Sindhu, Lana H. Gimber, Lee Cranmer, Ali McBride, Andrew S. Kraft

**Affiliations:** 1 Department of Medicine, Division of Hematology/Oncology, The University of Arizona, 1501 N Campbell Ave, Tucson, AZ 85724 USA; 2 Department of Medical Imaging, The University of Arizona, 1501 N Campbell Ave, Tucson, AZ 85724 USA; 3 Seattle Cancer Care Alliance, 825 Eastlake Ave, Seattle, WA 98109 USA; 4 The University of Arizona Cancer Center, Suite 2912, 1515 N Campbell Ave, Tucson, AZ 85724 USA

## Abstract

**Background:**

Angiosarcomas are tumors of malignant endothelial origin that have a poor prognosis with a five-year survival of less than 40%. These tumors can be found in all age groups, but are more common in older patients; with the cutaneous form most common in older white men. Combined modality therapy including surgery and radiation appears to have a better outcome than each modality alone. When metastatic, agents such as liposomal doxorubicin, paclitaxel and ifosfamide have activity but it is short-lived and not curative. Immunotherapy targeting either the PD-1 receptor or PD-L1 ligand has recently been shown to have activity in multiple cancers including melanoma, renal, and non-small lung cancer. Although these agents have been used in sarcoma therapy, their ability to treat angiosarcoma has not been reported.

**Case presentation:**

Here we describe the case of a 63-year-old man who presented initially with angiosarcoma of the nose and received surgery for the primary. Over 4 years he had recurrent disease in the face and liver and was treated with nab-paclitaxel, surgery, and radioembolization, but continued to have progressive disease. His tumor was found to express PD-L1 and he received off-label pembrolizumab 2 mg/kg every 21 days for 13 cycles with marked shrinkage of his liver disease and no new facial lesions. Secondary to this therapy he developed hepatitis and has been treated with decreasing doses of prednisone. During the 8 months off therapy he has developed no new or progressive lesions.

**Conclusions:**

Although occasional responses to immunotherapy have been reported for sarcomas, this case report demonstrates that angiosarcoma can express PD-L1 and have a sustained response to PD-1 directed therapy.

## Background

Angiosarcomas are complex soft-tissue sarcomas that are aggressive often based on malignant endothelial origin involving blood and lymph vessels. Approximately 2 % of soft tissue sarcomas and 5 % of cutaneous sarcomas are diagnosed as angiosarcomas [1]. The incidence of angiosarcoma has risen over the last several decades with a higher prevalence in older Caucasian males with average age at diagnosis of 65–70 [1, 2]. The prognosis of these tumors is poor with a reported 5-year survival rate of less than 40%.

Current treatment includes surgery with wide-field radiotherapy; however, the tumor tends to invade tissue and is often prone to incomplete excision [3]. Studies have reported success with a combined-modality approach of surgical resection followed by postoperative radiation therapy and/or chemotherapy [4]. A recent retrospective study evaluated survival outcomes of 55 patients with angiosarcoma of the face and scalp treated with either single modality or multimodality therapy with surgery, radiation and/or chemotherapy [5]. Patients who underwent multimodality treatment had significantly favorable local regional control (20% vs 11%; *P* = .04), recurrence-free survival (19% vs 10%; *P* = .02) and higher overall survival (46% vs 16%; *P* = .04) when compared with patients treated with single modality treatment [5]. Even after optimal local-regional treatment, patients are still at risk for the development of distant metastases [1–3] Doxorubicin-based regimens, taxanes and ifosfamide as single agents or in combination regimens are used to treat metastatic angiosarcoma with PFS ranging from a median of 3.7 to 9.5 months. One study reported an overall survival with weekly paclitaxel of 7.6 months [2].

The immune system is critical in cancer control and progression, and appropriate modulation of the immune system may provide an effective therapeutic option for sarcoma. Early observations in patients with renal transplant showed that patients developed Kaposi’s sarcoma at a higher rate than the control population implying that the immune system can play a role in the natural history of this disease [6]. In addition, in a study by Penn and colleagues evaluating 8191 patients that had both organ allografts and immunosuppression they found that 1.7% of patients developed sarcoma, a higher incidence of sarcoma compared to the general population [7]. Results of an adjuvant immunotherapy for pediatric sarcomas suggested that overall survival in these patients was increased, although a double blind study was not carried out [8]. This finding suggested a role for the immune system in regulating sarcoma outgrowth. The recent success of immune checkpoint inhibitors that target either PD-1 or PD-L1 in treatment of melanoma, non-small cell lung, renal and bladder carcinomas suggests that immunotherapy might play an important role in sarcoma therapy.

Here we describe a 63-year-old man with refractory metastatic cutaneous angiosarcoma who obtained an ongoing major partial response with the PD-1 inhibitor pembrolizumab after failure of surgery and chemotherapy to control his disease.

## Case presentation

**Fig. 1:**
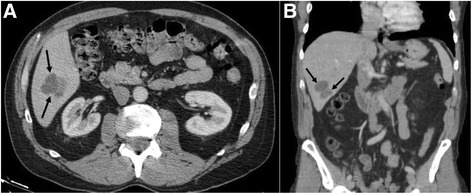
**a** Axial and **b** coronal post-contrast CT images through the abdomen before the start of treatment with evofosfamide demonstrates an index lobulated hypodense lesion (*arrows*) within the right hepatic lobe consistent with metastatic disease

**Fig. 2:**
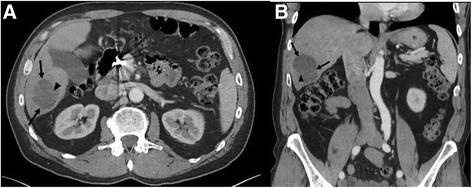
**a** Axial and **b** coronal post-contrast CT images through the abdomen approximately 3 months status post Yttrium-90 radioembolization and after the start of evofosfamide clinical trial demonstrate the index metastatic lesion (*arrows*) within the right hepatic lobe with expected post-treatment changes and internal areas of hemorrhage (*arrow heads*), however without a significant decrease in size

A 63-year-old Caucasian male with was initially diagnosed with angiosarcoma of the nose in October 2011 and underwent rhinectomy with negative margins followed by 3 reconstructive operations using a forehead flap. The patient also had a medical history of chronic lymphocytic leukemia which was untreated and being observed. In September 2012, the patient noticed two new lesions, one on his left cheek and the other on his right submandibular region and surgical resection occurred to remove these lesions in December 2012. Adjuvant treatment ensued with nab-paclitaxel dosed at 100 mg/m^2^ weekly on a 28-day cycle starting in February 2013 for 2 cycles. In November 2013, CT of the chest and abdomen demonstrated multiple new hepatic lesions (Fig. 1) and the patient was started on clinical trial with evofosfamide (TH-302) and received 4 cycles of treatment. The patient progressed on study and was referred to interventional radiology where he underwent bilobar radioembolization with Yttrium-90. Repeat CT examination following Yttrium-90 radioembolization demonstrated post treatment changes without significant decrease in size of the hepatic lesions (Fig. 2). However, in December 2014 the patient developed a new right jaw mass and was started on another cycle of nab-paclitaxel.

**Fig. 3:**
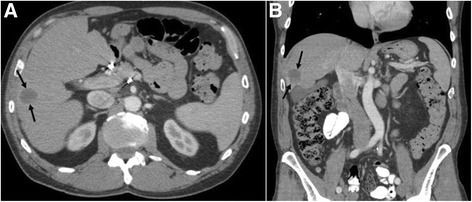
**a** Axial and **b** coronal post-contrast CT images through the abdomen during PD-1 therapy again demonstrate the index metastatic lesion (*arrows*) within the right hepatic lobe with post-treatment changes and marked decrease in size

**Fig. 4:**
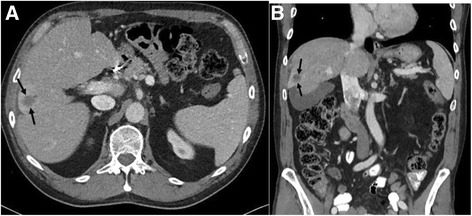
**a** Axial and **b** coronal post-contrast CT images through the abdomen after completing 13 cycles of immunotherapy and despite being off treatment for 4 months secondary to hepatitis again demonstrate the index metastatic lesion (*arrows*) within the right hepatic lobe with continuing decrease in size

The patient had noticeable disease progression with interval development of a right tongue mass. Staining of the patient’s tumor tissue with Roche SP142 revealed positive PD-L1 expression a measured by greater than 5% of the tumor cells staining positive. In 2015, based on the positive tumor expression, off-label treatment with pembrolizumab dosed at 2 mg/kg every 21 days was begun. The patient had concurrent radical excision of the right jaw soft tissue angiosarcoma with advanced flap closure. Subsequent restaging CT scans demonstrated a significant response of the liver lesion (Fig. 3). After completion of 13 cycles of pembrolizumab he developed autoimmune hepatitis. The immunotherapy was held and the patient was started on prednisone with a step down taper. In March 2016, while on prednisone therapy, he had another CT study of the abdomen/pelvis (Fig. 4) that revealed a further decrease in size of the liver disease. Additional body scans and biopsy did not detect any new disease at this time.

## Discussion

Angiosarcoma treatment often includes combined radical surgical excision and radiotherapy. However, over 60% of patients ultimately develop distant metastases. In the metastatic setting, chemotherapy rarely leads to complete and durable responses; therefore, there is an unmet clinical need for more effective therapies to induce durable responses. In the last 2 years, the advent of immunotherapy has provided a promising alternative for classical cytotoxic chemotherapy. Sensitivity to immunotherapy is thought to be secondary to higher DNA mutation rates, for example those found in melanoma and non-small cell lung cancer. While in comparison the mutation rate in acute leukemia is low. The genetic aberrations involved in the development of angiosarcoma are still poorly understood. Until recently, most of the genetic alterations were identified mostly in secondary but not in primary angiosarcomas. The most frequent chromosomal abnormalities were on chromosome 8q, 10p and 5q [9]. Additonally, MYC amplification is a recurrent genetic mutation in secondary angiosarcoma. Recently, Shon and his colleagues confirmed the presence of both MYC amplification and MYC overexpression in a minority of primary angiosarcomas [10]. In their study, by IHC analysis, MYC overexpression was present in 9 of 38 (24%) cases. Six of the nine positive cases were from the head and neck region [10]. Therefore, in comparison to other tumor types, angiosarcoma would appear to have an intermediate mutation frequency.

The anti-CTLA4 antibody ipilimumab has been given to six patients with synovial sarcoma without a response [11]. Current data using PD-1 inhibitors for the treatment of sarcoma is limited. PD-1 and PD-L1 expression has been reported in a variety of sarcomas, although the limited number of angiosarcomas tested have been negative for this marker. One study reviewed PD-1 and PD-L1 expression in 105 patients with multiple subtypes of sarcoma and found that intratumoral infiltration of PD-1 positive lymphocytes was seen in 65% of cases and PD-L1 tumor expression in 58% [12]. PD-1 and PD-L1 expression was correlated with higher tumor grade, advanced stage of disease, presence of metastasis, and poor clinical outcome [12]. Another study evaluated various subtypes of soft tissue in 50 sarcoma patients, and pathologic expression revealed lower levels of PD-L1 expression on tumor cells and infiltrating lymphocytes (12% and 30%, respectively) [13]. In this study, there was no association between clinical features and overall survival with PD-L1 expression in tumor or immune infiltrates. The authors proposed that the reason for the differing results could be due to the small heterogeneous cohort and the use of a differing PD-L1 assay in these studies.

Despite the lack of a large clinical trial data showing definitive efficacy in sarcomas, there is data from smaller trials that show that immunotherapy has activity against certain sarcomas. Tawbi and colleagues evaluated the use of pembrolizumab in soft tissue sarcoma and bone sarcoma in a phase II study [14]. Five of the 76 patients had a partial response after 20 weeks of treatment suggesting that the use of PD-1 and PD-L1 blockade does show efficacy in the treatment of sarcomas and may provide a viable treatment option for patients [14]. Another retrospective analysis included 23 metastatic soft tissue sarcoma or bone sarcoma patients treated with nivolumab [15]. Thirteen of these patients also received concomitant treatment with the tyrosine kinase inhibitor, pazopanib. Patients had baseline imaging and then restaging imaging after 6 cycles of treatment and 9/23 patients were found to have clinical benefit, with either a partial response or stable disease [15].

## Conclusion

Our patient also has an underlying history of indolent CLL for which he never received treatment. Although there is no evidence that patients with an underlying B cell lymphoproliferative disorder respond better to treatment with PD-1 and PD-LI inhibitors, given the increased number of B cells and their potential regulation of CD8 T cells, it is possible that this may have contributed to his response and warrants further evaluation. This case report demonstrates a clear response of angiosarcoma to a PD-1 inhibitor and the potential for the use of these inhibitors in this disease.
